# Magnetostrictive and Magnetoactive Effects in Piezoelectric Polymer Composites

**DOI:** 10.3390/nano14010031

**Published:** 2023-12-21

**Authors:** Oleg V. Stolbov, Yuriy L. Raikher

**Affiliations:** 1 Laboratory of Dynamics of Disperse Media, Institute of Continuous Media Mechanics, Russian Academy of Sciences, Ural Branch, 614018 Perm, Russia; sov@icmm.ru; 2 Research and Education Center “Smart Materials and Biological Applications”, Immanuel Kant Baltic Federal University, 236041 Kaliningrad, Russia

**Keywords:** magnetoelectric, piezoelectric, magnetostrictive, magnetoactive, composites, polymer films

## Abstract

A mesoscopic model for a polymer-based magnetoelectric (ME) composite film is developed. The film is assumed to consist of a piezoelectric polymer matrix of the PVDF type filled with CFO-like single-domain nanoparticles. The model is treated numerically and enables one to obtain in detail the intrinsic distributions of mechanical stress, polarization and electric potential and helps to understand the influence of the main configurational parameters, viz., the poling direction and the orientational order of the particle magnetic anisotropy axes on the electric response of the film. As the model is fairly simple—it uses the RVE-like (Representative Volume Element) approach with a single-particle cell—the results obtained are rather of qualitative than quantitative nature. However, the general conclusions seem to be independent of the particularities of the model. Namely, the presented results establish that the customary ME effect in composite films always comprises at least two contributions of different origins, viz., the magnetostrictive and the magnetoactive (magnetorotational) ones. The relative proportion between those contributions is quite movable depending on the striction coefficient of the particles and the stiffness of the polymer matrix. This points out the necessity to explicitly take into account the magnetoactive contribution when modeling the ME response of composite films and when interpreting the measurements on those objects.

## 1. Introduction

The essence of the functionality of magnetoelectric (ME) composites is the mediating role of mechanical stresses. Along this route either the magnetic phase acts on the piezoelectric one (the direct ME effect) or vice versa (the inverse ME effect). From that stem all the versatile applications of ME transducers and convertors as sensors and actuators [1,2], non-volatile memory [3], energy harvesters [4,5,6], activators of tissue regeneration processes [7,8,9,10,11], etc.

### 1.1. Magnetostrictive Composites

In the conventional paradigm, the mechanical link in the ME transformation is established by the magnetostriction effect, i.e., the change of dimensions of a ferromagnetic object under magnetization. This equally relates to solid two-component systems like ferromagnet (FM)+piezoelectric (PE) and to two- and three-component polymeric compositions of the FM+polymeric PE or FM+PE polymer+solid PE types. Therefore, to make the conversion efficient, the magnetic powders of the substances with high magnetostriction coefficients are used, first place, cobalt ferrite Co ${}_{1-x}$Fe${}_{2+x}$O${}_{4}$ (CFO) and Terfenol-D.

Typical solid PE materials used in the ME composites are ceramics BaTiO${}_{3}$ (BTO) and Pb[Zr${}_{x}$Ti${}_{1-x}$]O${}_{3}$ (PZT). The most popular polymeric piezoelectrics are PVDF (poly-vinylidene fluoride) and its co-polymers. A substantial part of their supramolecular structure consists of the crystallite β phase that displays high piezoelectric response comparable to that of customary solid piezoelectrics [12,13,14]. To align the electric polarization directions in the β-phase domains, the PVDF films are subjected to the poling procedure and are worked on with a high-strength electric field.

When producing PVDF-based composite films, CFO nanoparticles are added to the yet non-solidified polymer, then the mixture is stirred up to homogenize and finally dried, see [15,16,17,18], for example. The working conditions for such *magnetostrictive* films are set with the aid of a constant magnetic (bias) field $H_{0}$. Under the action of $H_{0}$, the ferrite particles change their shapes and become the sites of local (mesoscopic) mechanical stresses. Those stresses either directly act on the surrounding PE phase (a two-component composite) or perturb the polymeric matrix that, in turn, transfers the stress to the embedded PE particles (a three-component composite).

In any case, the applied field $H_{0}$ induces a stationary electric polarization that manifests itself as the transverse voltage difference Δφ between the film faces. This state is used as the operating point of an ME device, the bias strength is chosen in such a way that the steepness of function $d\lambda /dH_{0}$ was maximal; here λ is the coefficient of linear magnetostriction that characterizes the field-induced FM particle strain. Such a composite responds to an applied probing field $H_{t}$ by generating an electric signal: the voltage difference at the film changes by increment δφ. In general, the magnitude of δφ depends nonlinearly on the bias field as the magnetostriction is saturated under a strong field. However, for $H_{t}\;\ll H_{0}$ the electric response is linear in $H_{t}$. Because of that, the efficiency of ME conversion is conventionally expressed in terms of coefficient $\alpha_{V}=\delta \varphi /\left(\ell \;\cdot \;H_{t}\right)$ which is a specific ME ‘susceptibility’ of the film; here *ℓ* is the film thickness.

### 1.2. Magnetoactive Composites

A particle of a magnetically-hard ferrite (CFO, for example) possesses a permanent magnetic moment μ of its own, and due to that any field H that is non-collinear with μ creates a torque which, having been transferred to the particle anisotropy axis, strives to turn the particle body in such a way that μ aligns with H. In such a situation, a particle that dwells inside a composite produces mechanical stresses in its surroundings. These stresses, however, have nothing to do with the magnetostriction effect as the particle shape does not change. Let us term this way of generating internal stresses as *magnetoactive* using the analogy with the magnetoactive polymers.

Therefore, in a magnetoactive composite, the origin of the electric signal is the same as in the magnetostrictive one, but the origin of the stress is different. As we have shown [19], the ME effect in a three-component magnetoactive composite but weakly depends on the elastic modulus of the matrix, so the latter might be varied in a wide range. On the other hand, the properties of three-component composites strongly depend on the mutual positions of the FM and PE particles. This structural “hypersensitivity” is absent in a two-component system of the FM/PVDF kind, and because of that, such systems are more appropriate for making magnetoactive composites.

Looking into the literature, one finds but few examples of the systems which might be considered magnetoactive or the prototypes of those. The more interesting is the case reported in Ref. [20] where the ME effect was studied in a dispersion of feroxyhyte δ-Fe${}^{3+}$O(OH) nanosheets in PVDF-TrFE. Although feroxyhyte, being a ferrohydroxide, is but weakly magnetic, it is remarkable by the virtual absence of magnetostriction. Therefore, in the composite under study, the conventional ME mechanism was totally “switched-off”. As the magnetoelectric effect in this system was observed and measured, this means that in Ref. [20] the existence of the magnetoactive way of ME conversion has been demonstrated experimentally. A short discussion of that notable work and the essence of the effect discovered there might be found in Ref. [19].

Carrying on this line of considerations, one would suppose that the magnetoactive mechanism, in fact, should have rather significantly contributed (certainly, together with the magnetostrictive one) to the ME effect in the PVDF films filled with other anisometric objects: nanoplatelets of barium ferrite [21,22], metal hydroxide Co(II)Fe(III)-O(OH) [23], and metal nanowires [24].

## 2. Coexistence of the Magnetostrictive and Magnetoactive ME Effects

It follows that in any ME composite both effects—the magnetostrictive as well as the magnetoactive one—should coexist. Meanwhile, in the literature on composite polymer magnetoelectrics, both on research and on the technology of those materials, the focus of attention is exclusively set on the magnetostriction mechanism, see the reviews [5,25,26,27,28,29,30], for example. This is no surprise as those contributions are rather difficult to distinguish from one another in the measurement results.

The goal of the present work is to carry out, by means of numerical modeling, a comparative analysis of the above-described effects. For that, the following model system is used. A composite film consists of a polymer matrix with the properties of PVDF that is filled with fine spherical particles with the properties of CFO. The polar nature of the PVDF molecules ensures their strong adhesion to the particles, so that the latter may be treated as tightly “glued” to the matrix. Due to that, any shape or position changes of the particles induce stresses in the matrix (note that the accompanying deformations might be quite small) and by that create piezoelectric polarization. The matrix is assumed to have passed the poling procedure and, hence, is characterized by a “global” unit vector ν of piezoelectric anisotropy. The FM nanoparticles are single-domain but sufficiently large to be free of superparamagnetism. Given that, for their description, one may employ the Stoner–Wohlfarth model with uniaxial magnetic anisotropy of the easy-axis type whose direction is described by unit vector n.

Under the application of magnetic field H that is non-collinear with n, the particle changes its shape, and by that induces the magnetostrictive part of the mechanical stress. Simultaneously, the magnetoactive part turns up as well. Indeed, at H=0 the particle magnetic moment μ points along one of the directions of the anisotropy axis: μ‖n. As a single-domain particle is magnetized to saturation, its magnetic susceptibility along n is zero; this implies that the susceptibility tensor of the particle may be presented in the form

(1) $$\chi_{ik}=\chi \left(\delta_{ik}-n_{i}n_{k}\right).$$

In the field H whose strength is below the lowest coercive force $K/M_{s}$ of the Stoner–Wohlfarth particle—here *K* is the anisotropy constant and $M_{s}$ saturation magnetization of the ferrite—the particle would magnetize according to

(2) $$M_{i}=M_{s}n_{i}+\chi_{ik}H_{k}=M_{s}\left[n_{i}+\frac{H_{k}}{H_{A}}\left(\delta_{ik}-n_{i}n_{k}\right)\right],$$

where $H_{A}=2K/M_{s}$ is the reference value of the anisotropy field. The torque that the particle exerts on the matrix is

(3) $$\left(\mu \times H\right)=M_{s}Hv\left(n\times h\right)\left[1-\frac{H}{H_{A}}\left(nh\right)\right];$$

here μ=Mv is the magnetic moment of a single-domain particle, *v* is its volume, h=H/H is the unit vector of the field. Note that expressions (2) and (3) are valid to the first order in parameter $H/H_{A}$, that is, our consideration applies to the field range $H<\frac{1}{2}H_{A}=K/M_{s}$.

## 3. Superposition of the Effects: Qualitative View

**Figure 1:**
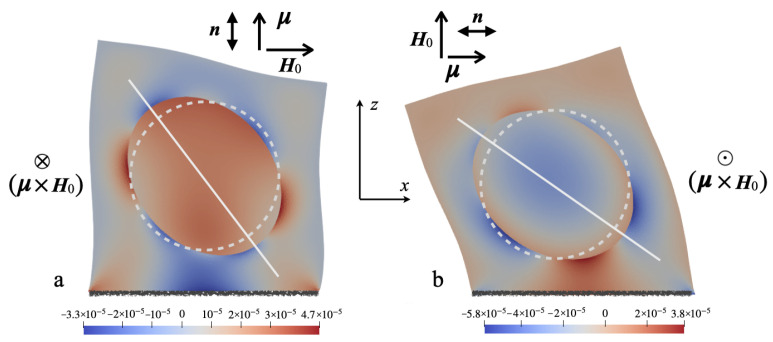
A 2D cell that comprises a single-domain uniaxial particle experiencing the joint action of magnetostrictive and magnetorotational effects; white lines mark the direction of the long axis of the striction-deformed particle. The color renders the shear stress distributions, the corresponding strains are enhanced by $10^{5}$ against the spatial scale of the figure. Panels (**a**) and (**b**) differ by the relative positions of μ and $H_{0}$ vectors and, hence, the direction of the magnetic torque.

To obtain a qualitative notion of how the magnetostrictive and magnetoactive (magnetorotational) mechanisms manifest themselves in joint action, we consider a 2D particle sitting inside a square cell filled with an elastic continuum; one of the cell sides is made immovable. In Figure 1 shear stress distributions for two variants of mutual orientations of the particle magnetic moment μ and applied field $H_{0}$ evaluated according to the procedure described in Section 5 are shown. The performed calculation employed the set of material parameters typical for the system CFO/PVDF; however, for the sake of visualization, the obtained values of strain are magnified by several orders. This modification reveals that magnetostriction deforms the particle: being initially a sphere it becomes prolate. Note, that in both panels, the acquired shapes of the particle are the same; this is because the magnetostriction effect does not depend on the absolute position of vectors n and $H_{0}$ in the plane passing through them. On the other hand, the magnetorotational effect preserves the particle shape; it just strives to align the magnetic moment, together with the anisotropy axis, with the field direction. Unlike the previous one, in this response mode, the sign of the effect inverts depending on the relative orientation of vectors μ and $H_{0}$. From that one concludes that, given the positions of the reference vectors, the two above-mentioned effects may either counteract one another (Figure 1a) or act synergistically (Figure 1b).

## 4. Energy Functional of the Composite Film

**Figure 2:**
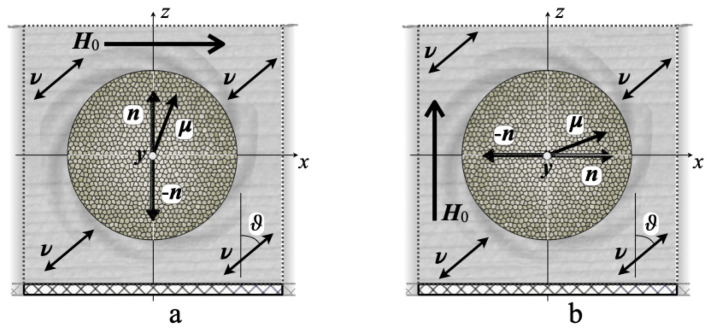
Schematic cross-sections of the elementary cubic cell for two variants of the initial position of the particle; panels (**a**) and (**b**) differ by the orientation of the applied field and magnetic anisotropy axis of the particle relative to the film plane; angle ϑ denotes the poling direction ν.

The model object under consideration is a film made of a composite of the PVDF/CFO type. The film is constructed as an infinite layer of elementary representative cells rigidly fixed on a solid plane. The representative cell—two variants are shown in Figure 2—is a cube of edge *ℓ* at the center of which there is positioned a spherical magnetically hard particle of radius $R_{p}$ the direction of whose magnetic anisotropy easy axis is defined by the unit vector n. The rest of the cell is filled with a PVDF-like polymer that had been subjected to poling that imparted to it a piezoelectric anisotropy characterized by the unit vector ν; the lower (z=0) face of the cell is immovable. Inside the layer, the cells are coupled by means of periodic boundary conditions imposed on all the basic thermodynamic variables, viz., mechanical stresses and strains and magnetic and electric fields. All the layer is embedded in the computational box whose dimensions in the Oz direction are far greater than the film thickness. In this scheme, the average variables evaluated for a single cell coincide with those of the whole layer.

The 3D problem of evaluating the state of the representative cell is formulated in terms of the finite-strain theory. In that approach, the deformation of a body is described as its transition from the initial state to an actual one. Accordingly, a point whose position in the initial state was given by radius-vector r, in the actual state, i.e., after deformation, is positioned at R=r+u, where u is the displacement vector. In this framework, the role of the spatial derivative is allotted to the deformation gradient $F=I+{\left(\nabla u\right)}^{T}$ whereas the strain tensor is defined as $E=\frac{1}{2}\left(F^{T}\cdot F-I\right)$ where I is the unit tensor. Further details are given in Appendix A below.

For the modeling, the representative cell in the initial state is presented as a sum of spatial regions: $\Omega_{m}^{\left(0\right)}$ and $\Omega_{p}^{\left(0\right)}$ occupied by the FM particle and the polymer matrix, respectively. Besides that, the cell is embedded in the computational box $\Omega^{\left(0\right)}$ whose bounds along directions ±Oz are positioned at a distance that is far greater than *ℓ*.

As the derivation of the energy functional of the film is quite cumbersome, its details are given in Appendix A. The resulting formulas are as follows

(4) $$U=U_{\mathrm{magn}.\mathrm{el}}+U_{\mathrm{elast}},$$

where the magnetic/electric part is

(5) $$U_{\mathrm{magn}.\mathrm{el}}=\int_{\Omega_{m}^{\left(0\right)}}\left(W_{\mathrm{magn}}+W_{\mathrm{elec}.m}\right)JdV_{0}+\int_{\Omega_{p}^{\left(0\right)}}W_{\mathrm{elect}.p}\;JdV_{0}-\frac{1}{8\pi }\int_{\Omega^{\left(0\right)}}\left(H^{2}+E^{2}\right)JdV_{0},$$

with J=det(F) being the Jacobian and $dV_{0}$ the volume element in the initial configuration. The explicit forms of the energy densities $W_{\mathrm{magn}}$, $W_{\mathrm{elec}.m}$ and $W_{\mathrm{elec}.p}$ are rendered by Equations (A7) and (A9) in Appendix A; besides internal magnetic field H, those functions depend on the internal electric field E as well. The material parameters pertinent to $U_{\mathrm{magn}.\mathrm{el}}$ are as follows: $M_{s}$ is saturation magnetization of the ferrite, $H_{A}$ is the internal field of uniaxial magnetic anisotropy, $\lambda_{s}$ is the saturation value of magnetostriction coefficient, $\varepsilon_{m}$ and $\varepsilon_{p}$ are dielectric permeabilities of the ferrite and polymer, respectively. Also, the set of material parameters includes the piezocoefficients $d_{ik}$ relevant to PVDF.

Concerning the elastic part of the energy functional, we note that polymerized PVDF is a rather stiff material, so the strains produced inside the film by the FM particles driven by moderate magnetic fields, are quite small. This assumption justifies the hypothesis of additivity of elastic and inelastic strains that is essential here since both the magnetostrictive $e^{\left(\mathrm{strict}\right)}$ and piezoelectric $e^{\left(\mathrm{piezo}\right)}$ strains belong to the latter type: they do not contribute to the elastic energy [31]. The particular expressions for $e^{\left(\mathrm{strict}\right)}$ and $e^{\left(\mathrm{piezo}\right)}$ for the considered case are derived in Appendix B and Appendix C, respectively. The elastic part of the functional is

(6) $$U_{\mathrm{elast}}=\int_{\Omega_{m}^{\left(0\right)}}\;W_{\mathrm{elast}.m}\left(E-e^{\left(\mathrm{strict}\right)}\right)\;dV_{0}+\int_{\Omega_{p}^{\left(0\right)}}\;W_{\mathrm{elast}.p}\left(E-e^{\left(\mathrm{piezo}\right)}\right)\;dV_{0},$$

where the explicit forms of $W_{\mathrm{elast}.m}$ and $W_{\mathrm{elast}.p}$ are given in Appendix A, see Equations (A12) and (A13). The material parameters that enter those expressions are the Young moduli $E_{m}$ and $E_{p}$ for the ferrite and polymer and their Poisson coefficients $\nu_{m}$ and $\nu_{p}$.

A necessary point when using the finite-element method is that displacement u should be continuous everywhere in $\Omega^{\left(0\right)}$. For that, to the space $\Omega_{s}^{\left(0\right)}=\Omega^{\left(0\right)}\setminus \left[\Omega_{m}^{\left(0\right)}\cup \Omega_{p}^{\left(0\right)}\right]$ that surrounds that film, one ascribes an elastic potential of the same Saint–Venant–Kirchhoff functional form as in Equations (A12) or (A13). However, the Young modulus $E_{s}$ of that “virtual” material is set several orders of magnitude lower than the real moduli $E_{p}$ and $E_{m}$. Upon that, the particular value of $E_{s}$ does not affect the results of the modeling. In accordance with this requirement, in our calculations instead of $U_{\mathrm{elast}}$ as such, a modified expression is used:

(7) $$U_{\mathrm{elast}}^{\prime }=U_{\mathrm{elast}}+\int_{\Omega_{s}^{\left(0\right)}}W_{\mathrm{elast}.s}\;dV_{0}.$$

Therefore, to describe the state of the considered representative cell, one has to minimize the energy functional

(8) $$U=U_{\mathrm{magn}}+U_{\mathrm{elast}}^{\prime }$$

defined by Equations (6)–(8) and the pertinent formulas from Appendix A, Appendix B and Appendix C. In other words, one has to solve variational equation

(9) $$\delta U=\frac{\partial U}{\partial (\nabla u)}\;\cdot \;\cdot {\left(\nabla \delta u\right)}^{T}+\frac{\partial U}{\partial \nabla \psi }\;\cdot \;\nabla \delta \psi +\frac{\partial U}{\partial \nabla \varphi }\;\cdot \;\nabla \delta \varphi =0,$$

where u is the displacement vector, whereas ψ and ϕ are scalar magnetic and electric potentials, respectively; here the dots denote scalar multiplication.

Before proceeding to calculations, Equation (9) is reduced to nondimensional form. For that a scaling factor *g* that is of the order of a reference value of non-zero piezotensor component: $g∼|\gamma_{i,kl}\left|∼\right|d_{ik}|$; in our calculations, we set $g=10^{-5}$ CGS units. In this representation, the variables and material parameters of the problem transform as

(10) $${\bar{d}}_{ik}=d_{ik}/g,\;\bar{H}=gH,\;\bar{E}=gE,\;\mathrm{etc}.;$$

the unit of distance is equal to the particle radius $R_{p}$ Under this choice, all the numerical coefficients in expression (9) and the equations it is based on, fall inside the interval [0.1÷10] that substantially enhances the stability of calculations.

**Figure 3:**
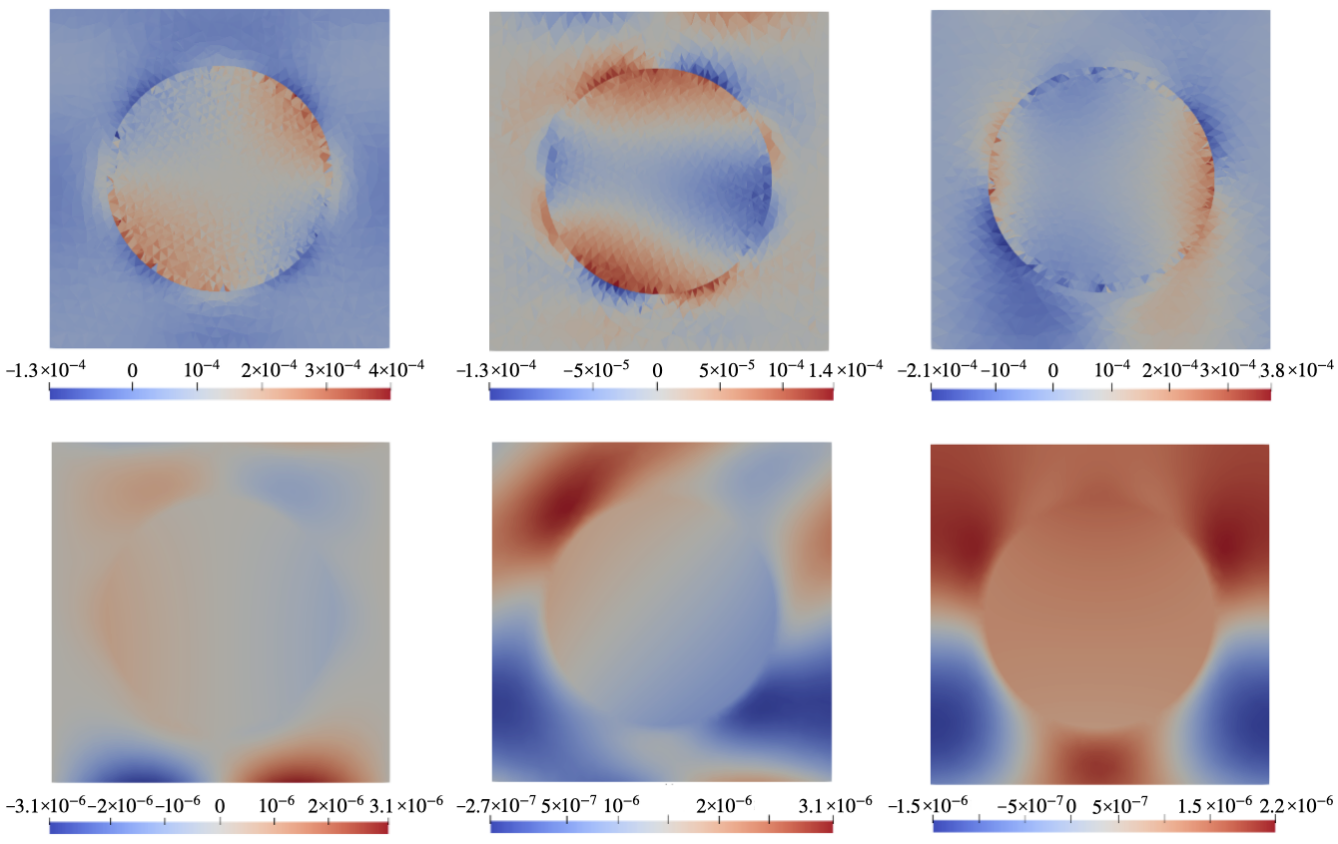
Color maps of mechanical stress **σ** and electric potential φ in xOz plane of the cell with configuration A (Figure 2a) under field $H_{0}=0.01$ directed along Ox for a CFO-like particle with $\lambda_{s}=-220$ ppm. **Top row**: components $\sigma_{xz}$ (**left**), $\sigma_{xx}$ (**center**), $\sigma_{zz}$ (**right**); **bottom row**: electric potential φ under poling directions $\vartheta =0^{\circ }$ (**left**), $45^{\circ }$ (**center**), $90^{\circ }$ (**right**).

**Figure 4:**
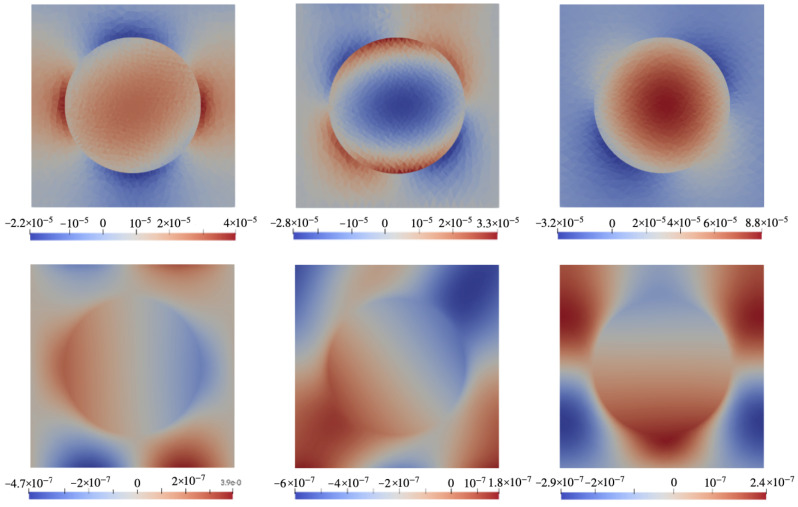
The same as in Figure 3 for a ferromagnet particle with $\lambda_{s}=0$.

**Figure 5:**
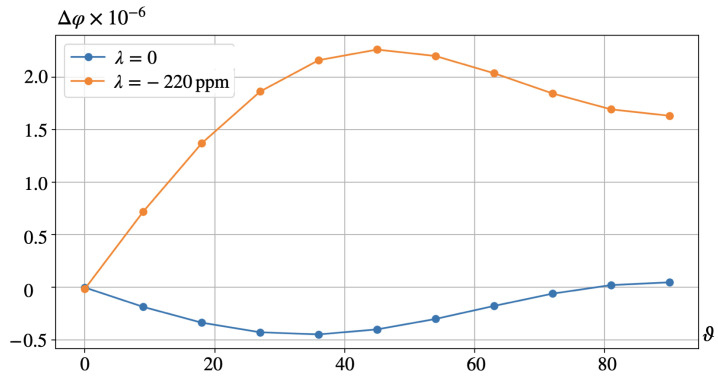
Configuration A, see Figure 2a. Dependence of the transverse voltage on the orientation of the piezoelectric axis ν for $\lambda_{s}=-220$ ppm and $\lambda_{s}=0$.

**Figure 6:**
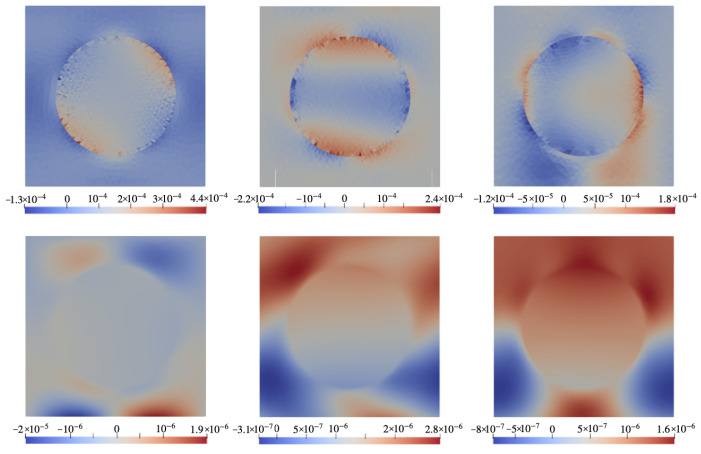
Color maps of mechanical stress **σ** and electric potential φ in xOz plane of the cell with configuration B (Figure 2b) under field $H_{0}=0.01$ directed along Oz for a CFO-like particle with $\lambda_{s}=-220$ ppm. **Top row**: components $\sigma_{xz}$ (**left**), $\sigma_{xx}$ (**center**), $\sigma_{zz}$ (**right**); **bottom row**: electric potential under poling directions $\vartheta =0^{\circ }$ (**left**), $45^{\circ }$ (**center**), $90^{\circ }$ (**right**).

**Figure 7:**
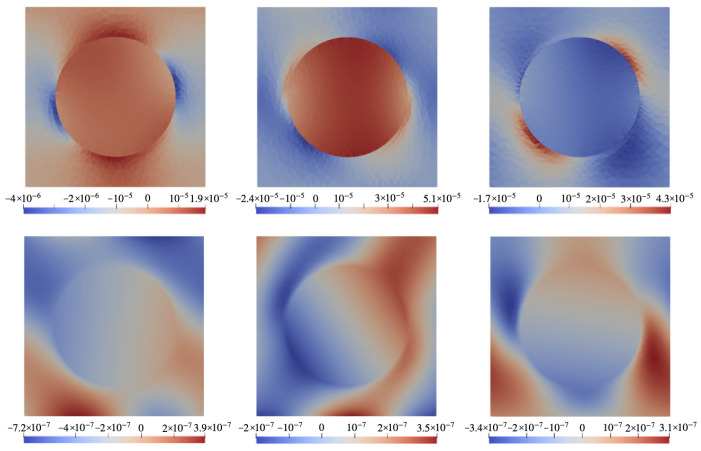
The same as in Figure 6 for a ferromagnet particle with $\lambda_{s}=0$.

**Figure 8:**
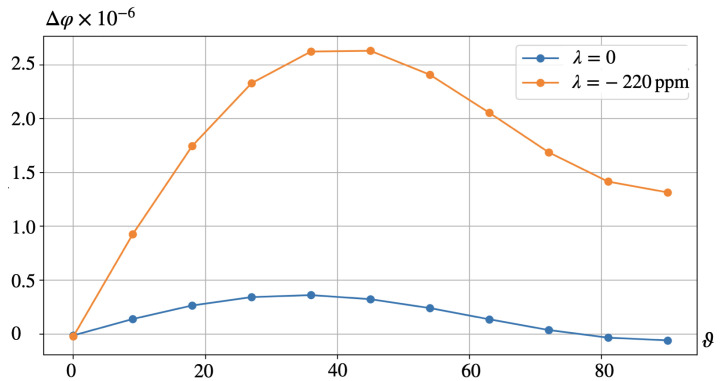
Configuration B, see Figure 2b. Dependence of the transverse voltage on the orientation of the piezoelectric axis ν for $\lambda_{s}=-220$ ppm and $\lambda_{s}=0$.

In below—in Figure 3, Figure 4, Figure 5, Figure 6, Figure 7 and Figure 8—all the results are given in the afore-introduced nondimensional units, the overline is omitted. The transition back to dimensional values is conducted at the very end of the consideration.

The particular values of material parameters used in our numerical calculations are as follows.

For CFO: $M_{s}=400$ Gauss, applied magnetic field $H_{0}=1$ kOe, anisotropy field $H_{A}=4$ kOe, reference magnetostriction coefficient $\lambda_{s}=220$ ppm, dielectric permeability $\varepsilon_{m}=100$, Young modulus $E_{m}=50$ GPa, Poisson coefficient $\nu_{m}=0.35$.
For PVDF: dielectric permeability $\varepsilon_{p}=10$, Young modulus $E_{p}=2$ GPa, Poisson coefficient $\nu_{p}=0.3$; the piezocoefficients are $d_{33}=-10^{-6}$, $d_{31}=5\times 10^{-7}$, $d_{15}=-7\times 10^{-7}$ in CGS units. The particle radius is set to R=15 nm.

## 5. Finite-Element Calculation

To find the minimum of the functional (8) with respect to unknown functions (u,ψ,φ), i.e., to solve Equation (9), the finite-element method is used in the realization FEM of package FEniCSx (written for python) that is an open-source computing platform for solving partial differential equations [32].

The calculation takes in the film element (representative cell) a cube with dimensions ℓ×ℓ×ℓ whose lower face is fixed. On all the sought-for functions, periodic boundary conditions along the Ox and Oy axes are imposed. Above and below the cell (along Oz) the cell abuts on right-angle prisms of high $h_{\mathrm{sp}}$; thus the calculation box (RVE proper) is a prism with cross-section ℓ×ℓ and of height $\left(\ell +2h_{\mathrm{sp}}\right)$.

The boundary conditions are

$$u{|}_{z=0}=0,\;\frac{\partial \psi }{\partial z}{|}_{z=-h_{sp},\ell +h_{sp}}=0,\;\frac{\partial \varphi }{\partial z}{|}_{z=-h_{sp},\ell +h_{sp}}=0,$$

that means that the lower face of the cell is fixed, and the derivatives of the sought-for functions zero out at the farthest boundaries of the calculation box.

## 6. Results

### 6.1. Configuration A

Figure 3 demonstrates the color maps of mechanical stresses and electric potential φ (defined against infinity) in the xOz cross-section of the cell of the configuration of Figure 2a) for a ferromagnet with the magnetostriction constant $\lambda_{s}=-220$ ppm. The top row shows the distributions of three components of the stress tensor in the field $H_{0}=0.01$ pointing along Ox. The bottom row presents the distributions of electric potential in the same plane for different orientations of the piezoelectric anisotropy axis (poling direction). Whereas the mechanical stresses do not depend on the direction of vector ν, the induced electric fields are essentially defined by that parameter since in each case the electric polarization is induced via different components of piezotensor d. Note that when evaluating internal magnetic fields, our calculation fully accounts for the demagnetizing fields inside the film.

Figure 4 is the analog of Figure 3 but for a particle made of a hypothetical ferromagnet with magnetostriction constant $\lambda_{s}=0$. As seen from the comparison, the distributions obtained for $\lambda_{s}\neq 0$ and $\lambda_{s}=0$ are drastically different. In this connection, it is important to note that Figure 3 presents the case of joint action of the magnetostriction and magnetic rotation of the particle whereas Figure 4 accounts for the case where only the magnetorotational effect takes part in generation of the electric response.

The bottom rows of Figure 3 and Figure 4 reveal that the distributions of electric potential φ strongly depend on the poling direction. A full view of this dependence is rendered by Figure 5 where not φ itself but the difference Δφ between the values of potential (averaged over the corresponding surface of the representing cell) at the opposite sides of the film is plotted. As it shows, the best results are attained when ν is oriented under $35÷45^{\circ }$ where both contributions are maximal. This takes place despite that the magnetorotational part (the lower curve) is negative in virtually all the angle intervals, reaching the maximum of about 20% of the magnetostrictive one. This evidences that a simple consideration presented in Figure 1a is indeed entirely correct in the qualitative aspect.

### 6.2. Configuration B

In Figure 6 the color maps rendering spatial distributions of stresses and electric potential (defined with respect to infinity) in the xOz cross-section of the representing cell in configuration B (see Figure 2b) for a system that contains a particle with magnetostriction constant λ=−220 ppm. The top row shows three main components of the stress tensor under field $H_{0}=0.01$ directed along Oz. The bottom row presents the spatial distribution of electric potential φ for different poling directions.

In case B, as in case A, Figure 7 is the analog of Figure 6 for a particle made of a hypothetical ferromagnet whose magnetostriction constant is identical zero. In this connection, we again note that Figure 6 presents the case of joint action of the magnetostriction and magnetic rotation of the particle whereas Figure 7 describes the case where the electric response is due solely to the magnetorotational effect. Quite expectedly, the bottom rows of Figure 6 and Figure 7, when compared, evidence that the distributions of electric potential φ obtained for the cases of $\lambda_{s}\neq 0$ and $\lambda_{s}=0$ are drastically different and depend strongly on the poling direction. A full view of this dependence is rendered in Figure 8 where the difference Δφ between the values of φ (averaged over the corresponding surface of the representing cell) at the opposite faces of the film is plotted. This figure shows that the best results correspond to the orientation range of ν from $35^{\circ }$ to $45^{\circ }$ where both contributions are positive and maximal; there the magnetorotational part makes about 20% of the magnetostrictive one. Recalling Figure 1, its panel (b), one finds that that simple illustration provides an entirely correct qualitative prediction.

## 7. Discussion

The results presented in Section 6 show that numerical simulations confirm the qualitative conclusions drawn in Section 2: depending on the magnetic and piezoelectric orientational textures established in the composite, the magentostrictive and magnetoactive (magnetorotational) mechanisms might either enhance or diminish their joint effect. Indeed, the magnetorotational contribution has opposite signs in A and B configurations whereas the magnetostrictive contributions are always positive. This explains why the obtained electric response Δφ in configuration B is about 20% higher than in that in configuration A.

It is instructive to compare the relative magnitudes of the two considered effects. A rough estimation may be deduced from comparing the magnetostrictive and magnetorotational torques that develop under the same applied field. We note that for small deviation angles, the magnetorotational torque is

(11) $$Q_{m.\mathrm{rot}}∼\left|\left(\mu \;\cdot \;H_{0}\right)\right|∼M_{s}vH_{0},$$

where *v* is the particle volume.

The magnetostrictve torque is produced by the particle shape change. From expression (A22) it follows that the magnetostriction strain under $H_{0}<\frac{1}{2}H_{A}$ is $e^{\left(\mathrm{strict}\right)}∼2\left(H_{0}/H_{A}\right)\lambda_{s}$. Then the energy excess and, thus, the torque which, due to magnetostriction, the particle exerts on the matrix is

(12) $$Q_{m.\mathrm{strict}}∼|e^{\left(\mathrm{strict}\right)}|E_{p}v∼\left|\lambda_{s}\right|E_{p}v.$$

Taking the ratio, one finds

(13) $$\xi \equiv Q_{m.\mathrm{rot}}/Q_{m.\mathrm{strict}}∼M_{s}H_{A}/2\left|\lambda_{s}\right|E_{p};$$

note that this estimation does not depend on the particle size.

The reference values for the material parameters used in our calculations are given in Section 4. Substituting these numbers in (13) one finds ξ∼0.2 that fairly well agrees with the results of numerical modeling presented in Figure 5 and Figure 8. However, as Equation (13) is based on rather rough assumptions, one should not overestimate the occurred closeness; much more important is that it yields a correct order of magnitude.

More important is that Equation (13) renders the parameter dependences which point out the relative roles of the effects. For instance, it predicts that under lower values of $\lambda_{s}$, like those for magnetite Fe${}_{3}$O${}_{4}$ ($\lambda_{s}∼17÷170$ ppm [33,34,35]) or NdFeB ($\lambda_{s}\approx 90$ ppm [36]), let alone barium ferrite BaFe${}_{12}$O${}_{19}$ ($\lambda_{s}\approx 9$ ppm [37]), the magnetorotational mechanism of generating the electric response may become fully comparable with the magnetostrictive one and even exceed the effect of the latter. The same enhancement might be encountered as well if to deal with softer polymer matrices, like those of specially prepared PVDF films whose Young moduli range 0.5÷1.3 GPa, see [14,38]. Besides that, under a fixed direction of the applied field, even the sign of the effect might change, see the curves in Figure 5.

Finally, we proceed to the magnitude of the modeled effects in dimensional form. The expressions of Section 4 yield $\Delta \bar{\varphi }=\bar{E}\;\cdot \;\bar{\ell }=\left(g/R\right)E\ell =\left(g/R\right)\Delta \varphi$. Setting the size of the magnetic particle to R≈15 nm and given $\Delta \bar{\varphi }∼2\;\times \;10^{-6}$, one finds

(14) $$\Delta \varphi ∼\left(R/g\right)\Delta \bar{\varphi }∼10^{-5}\;V\approx 100\;\mu V.$$

Using this for estimating the magnetoelectric susceptibility of the considered film, one obtains

(15) $$\alpha_{V}=\Delta \varphi /\left(\ell \;\cdot \;H_{0}\right)\approx 20\;\mathrm{mV}/\mathrm{cm}\;\cdot \;\mathrm{Oe}.$$

The obtained value is by no means a very high one if to just directly compare it to the scale of units or tens of volts attained in sensing and harvesting ME devices. However, when assessing this result of Equation (15), one has to put it in a different context. Indeed, the very statement of the above-solved problem applies not to the resonance regimes of cantilever-type setups but to quasi-static situations which are customary for experimental tests on stimulated cell development, i.e., tissue engineering.

Going along this line, we recall that in our simulations the volume fraction of CFO is $\phi_{v}\;∼\;0.15$ that, when recalculated to weight content for $\rho_{\mathrm{CFO}}\;∼\;5$ and $\rho_{\mathrm{PVDF}}\;∼\;2$ g/cm${}^{3}$, yields $\phi_{w}\;∼\;0.3$. If to consider a film with weight content $\phi_{w}∼0.1$ that is typical for the biologically-oriented ME films [39], one arrives at $\alpha_{V}∼7$ mV/cm·Oe. This value agrees well with the result reported in Ref. [39], where it had been found that a CFO/PVDF film with $\phi_{w}∼0.1$ yields $\alpha_{V}\approx 6.5$ mV/cm·Oe.

## 8. Conclusions

The major idea of our consideration is to emphasize that the ME effect in polymer composites with magnetically hard particles always comprises at least two contributions of different origins, namely, the magnetostrictive and the magnetoactive (magnetorotational) ones. Mesoscopic modeling seems an adequate way to justify this conclusion since it is capable of describing in detail the mechanical and electromagnetic fields that an applied magnetic field induces inside a composite. As a convenient example of a particular sample, a film is chosen due to its simple overall geometry. To make the calculation comparable, at least qualitatively, with experimental evidence, the ingredients of the model composite are ascribed the properties of a typical pair: the filler particles possess the material parameters inherent to CFO whereas the matrix piezoelectric and mechanical properties are those typical for PVDF. In our view, the results obtained confirm the basic idea of the coexistence of the two above-mentioned polarization-inducing effects. Besides, some signatures had been found in experiments but never accounted for explicitly. This makes it interesting to really measure those effects as separate contributions.

In connection with the subject under discussion, the idea of developing a purely magnetoactive (magnetorotational) magnetoelectric turns up and may be assessed. However, as our simulations predict such a prospect seems rather futile. Meanwhile, given that coexistence of the two above-mentioned ME effects is essential, it looks reasonable to use modelling for adjusting the content and texture of the composite in such a way that both effects would work in optimal proportion. Finally, the internal structure of the composite studied in the present work is too simple to be directly compared with the experiment in any quantitative way. On the other hand, the capabilities of the developed mesoscopic approach are evident. In our future work, we plan to gradually proceed with this toolbox to more complex problems of structural magneto-electro-mechanics of polymer composites.

## Data Availability

The datasets obtained and analyzed in the course of the presented study are available from the corresponding author on reasonable request.

## Appendix A. Derivation of the Energy Functional

In the finite-strain theory, to deal with the initial and actual configurations, the basis vectors are introduced as $\epsilon_{i}=\partial r/\partial q_{i}$ and ${\hat{\epsilon }}_{i}=\partial r/\partial q_{i}$ where $q_{i}$ denotes the spatial coordinates. The Hamiton operators (the analogs of the customary spatial derivatives) are defined as $\nabla =\epsilon^{\;i}\partial /\partial q_{i}$ and $\hat{\nabla }={\hat{\epsilon }}^{\;i}\partial /\partial q_{i}$, where $\epsilon^{\;i}$ and ${\hat{\epsilon }}^{\;i}$ are reciprocal basis vectors.

The fundamental kinematic tensor function (deformation gradient) has the form

(A1) $$F={\left(\nabla R\right)}^{T}={\hat{\epsilon }}_{i}\epsilon^{\;i}=I+{\left(\nabla u\right)}^{T},$$

where I is the metric (unit) tensor and index T denotes transposition. For the inverse function, one has

(A2) $$F^{-1}={\left({\hat{\nabla }}_{r}\right)}^{T}=\epsilon_{i}{\hat{\epsilon }}^{\;i}=I-\hat{\nabla }u^{T}.$$

In these notations, Hamilton operators in initial and actual configurations are related as follows:

(A3) $$\hat{\nabla }=F^{-T}\cdot \nabla .$$

In some cases, it is more convenient to use polar expansion of the strain gradient factorizing it as

(A4) F=O·U,

where O is an orthogonal rotation tensor and U is a symmetrical tensor of pure deformation, i.e., $O^{T}=O^{-1}$ and $U^{T}=U$.

The magnetic field is presented in the form $H=H_{0}-\hat{\nabla }\psi$, where ***H***_0_ is the external field and *ψ* scalar magnetic potential. Likewise, for the electric field, one sets $E=-\hat{\nabla }\varphi$ with *φ* being scalar electric potential. In accordance with the rules proposed in Ref. [40], we define the energy of a magneto-electro-elastic medium in the absence of currents. For the actual configuration, it is

(A5) $$\begin{array}{c} U=\int_{\Omega_{m}}\rho \phi_{\mathrm{magn}}\left(F,H,E\right)dV+\int_{\Omega_{p}}\rho \phi_{\mathrm{piezo}}\left(F,E\right)dV-\frac{1}{8\pi }\int_{\Omega }\left(H^{2}+E^{2}\right)dV; \end{array}$$

here ρ is the material density, $\Omega_{m}$ the region occupied by the FM particle and $\Omega_{p}$ that occupied by the matrix; $\phi_{\mathrm{magn}}\left(F,H,E\right)$ and $\phi_{\mathrm{piezo}}\left(F,E\right)$—magnetoelastic and electroelastic potentials define by Equation (A7) below.

Magnetization, polarization and the stress tensor are expressed as

(A6) $$\begin{array}{c} M=-\rho \frac{\partial \phi_{\mathrm{magn}}}{\partial H},\;P_{\mathrm{magn}}=-\rho \frac{\partial \phi_{\mathrm{magn}}}{\partial E},\;P_{\mathrm{piezo}}=\rho \frac{\partial \phi_{\mathrm{piezo}}}{\partial E},\\ \;P_{\mathrm{II}(\mathrm{magn})}=\rho_{0}\frac{\partial \phi_{\mathrm{magn}}}{\partial E_{\mathrm{elast}}},\;P_{\mathrm{II}(\mathrm{piezo})}=\rho_{0}\frac{\partial \phi_{\mathrm{piezo}}}{\partial E_{\mathrm{elast}}}; \end{array}$$

where $P_{\mathrm{II}}$ is the Piola–Kirchhoff stress tensor of second kind. Evidently, the electric polarizations and stress tensors are different inside different components of the composite.

The origin of the strain $E_{\mathrm{elast}}$ in the last two relations of the set (A6) is as follows. The full strain of the sample is rendered by the Green-Lagrange tensor $E=\frac{1}{2}\left(F^{T}\cdot F-I\right)$ that is split in two parts, elastic $E_{\mathrm{elast}}$ and inelastic $E_{n.\mathrm{elast}}$. The elastic strain is the contribution to the full strain that arises/disappears upon imposing/removal of mechanical load. The non-elastic part comprises the magnetostrictive strain (A22) of the FM particle that is derived in Appendix B, and the piezoelectric strain (A29) of the PE matrix that is derived in Appendix C.

In the general case, only the velocity form of the strain superposition holds $\dot{E}={\dot{E}}_{\mathrm{elast}}+{\dot{E}}_{n.\mathrm{elast}}$. However, provided the strains are small, one may remove the time derivatives and sum up those contributions as such. Moreover, in this limit, the Piola–Kirchhoff tensor reduces to the conventional Cauchy stress tensor **σ** whose components are presented in color maps of Figure 3, Figure 4 and Figure 6, Figure 7 and used below in Appendix C.

Functional (A5), when transformed to the initial configuration with the aid of relations $\rho =J^{-1}\rho_{0}$ and $dV=JdV_{0}$, where $dV_{0}$ and $\rho_{0}$ are volume element and mass density in the initial configuration and J=det(F), takes the form

$$U=\int_{\Omega_{m}^{\left(0\right)}}\rho_{0}\phi_{\mathrm{magn}}\left(F,H,E\right)dV_{0}+\int_{\Omega_{p}^{\left(0\right)}}\rho_{0}\phi_{\mathrm{piezo}}\left(F,E\right)dV_{0}-\frac{1}{8\pi }\int_{\Omega^{\left(0\right)}}J\left(H^{2}+E^{2}\right)dV_{0},$$

where the regions of integrations are transformed as well; for instance, $\Omega_{m}\Rightarrow \Omega_{m}^{\left(0\right)}$, etc.

In the actual configuration, magnetic and electric fields are also affected by the Hamilton operator yielding

$$H=H_{0}-F^{-T}\;\cdot \;\nabla \psi ,\;E=-F^{-T}\;\cdot \;\nabla \varphi .$$

Potentials $\phi_{\mathrm{magn}}$ and $\phi_{\mathrm{piezo}}$ in (A5) are defined as corresponding mass densities (for the FM and PE phases), each of which expands in a sum of magnetic/electric and elastic contributions:

(A7) $$\phi_{\mathrm{magn}}=\frac{W_{\mathrm{elast}.m}}{\rho_{0}}+\frac{W_{\mathrm{magn}}}{\rho }+\frac{W_{\mathrm{elec}.m}}{\rho },\;\phi_{\mathrm{piezo}}=\frac{W_{\mathrm{elast}.p}}{\rho_{0}}+\frac{W_{\mathrm{elec}.p}}{\rho }.$$

After these potentials are presented in explicit form, one may split functional *U* into magnetic/electric $U_{\mathrm{magn}.\mathrm{el}}$ and elastic $U_{\mathrm{elast}}$ parts. The first one is

(A8) $$U_{\mathrm{magn}.\mathrm{el}}=\int_{\Omega_{m}^{\left(0\right)}}\left(W_{\mathrm{magn}}+W_{\mathrm{elec}.m}\right)JdV_{0}+\int_{\Omega_{p}^{\left(0\right)}}W_{\mathrm{elect}.p}\;JdV_{0}-\frac{1}{8\pi }\int_{\Omega^{\left(0\right)}}\left(H^{2}+E^{2}\right)JdV_{0}.$$

The magnetic energy density is

(A9) $$W_{\mathrm{magn}}=-\frac{M_{s}}{J}\left\{\left(H\;\cdot \;O\;\cdot \;n\right)-\frac{1}{2H_{A}}\left[H^{2}-{\left(H\;\cdot \;O\;\cdot \;n\right)}^{2}\right]\right\},$$

so that for magnetization one has

(A10) $$M\left(H\right)=-\frac{\partial W_{\mathrm{magn}}}{\partial H}=\frac{M_{s}}{J}\left[O\;\cdot \;n-\frac{1}{H_{A}}\left(H-(H\;\cdot \;O\;\cdot \;n)O\;\cdot \;n\right)\right];$$

here O is the rotation operator defined above, and the dot denotes scalar multiplication.

The electric energy densities in the ferrite and polymer are rendered, respectively, by formulas

(A11) $$W_{\mathrm{elec}.m}=-\frac{\varepsilon_{m}-1}{8\pi }E^{2},\;W_{\mathrm{elec}.p}=-\frac{\varepsilon_{p}-1}{8\pi }E^{2};$$

where $\varepsilon_{m}$ and $\varepsilon_{p}$ are dielectric permeabilities of those materials; we assume them to be scalar quantities. In expressions (A11), vector E denotes the internal electric field that is in a standard way related to the polarization inside each medium:

(A12) $$P_{\mathrm{magn}}=\frac{\varepsilon_{m}-1}{4\pi }E,\;P_{\mathrm{piezo}}=\frac{\varepsilon_{p}-1}{4\pi }E.$$

Let us consider the part of the energy potential (A5) that renders the elastic strain contribution in the initial configuration

(A13) $$U_{\mathrm{elast}}=\int_{\Omega_{m}^{\left(0\right)}}\;W_{\mathrm{elast}.m}\left(E_{\mathrm{elast}}\right)\;dV_{0}+\int_{\Omega_{p}^{\left(0\right)}}\;W_{\mathrm{elast}.p}\left(E_{\mathrm{elast}}\right)\;dV_{0}.$$

To describe the mechanical properties of the film components, i.e., those occupying the regions $\Omega_{m}^{\left(0\right)}$ and $\Omega_{p}^{\left(0\right)}$, respectively, we use elastic potentials in the Saint–Venant–Kirchhoff form, see [41], for example:

(A14) $$W_{\mathrm{elast}.m}=\frac{E_{m}}{2(1+\nu_{m})}\mathrm{tr}\left(E_{\mathrm{elast}}^{2}\right)+\frac{\nu_{m}E_{m}}{2\left(1+\nu_{m}\right)\left(1-2\nu_{m}\right)}{\mathrm{tr}}^{2}\left(E_{\mathrm{elast}}\right),$$

(A15) $$W_{\mathrm{elast}.p}=\frac{E_{p}}{2(1+\nu_{p})}\mathrm{tr}\left(E_{\mathrm{elast}}^{2}\right)+\frac{\nu_{p}E_{p}}{2\left(1+\nu_{p}\right)\left(1-2\nu_{p}\right)}{\mathrm{tr}}^{2}\left(E_{\mathrm{elast}}\right),$$

The material parameters $E_{m}$ and $E_{p}$ in expressions (A14) and (A15) are the Young moduli of the ferrite and polymer, whereas $\nu_{m}$ and $\nu_{p}$ are their Poisson coefficients. That accomplished, the elastic part (A13) of *U* is fully determined.

## Appendix B. Magnetostriction of a Uniaxial Particle

In the lowest approximation in the powers of unit magnetization vector $m=M/M_{s}$, the volume density of magnetoelastic energy is presented in the form [31]:

(A16) $$u_{\mathrm{em}}=-\lambda_{iklm}\sigma_{ik}m_{l}m_{m},$$

where $\lambda_{iklm}$ is the tensor of magnetostriction coefficients, and $\sigma_{ik}$ is the mechanical stress tensor.

The condition of uniaxial symmetry implies that all the directions in the plane normal to the singled out axis, here it us n, are equivalent. Given that, tensor $\lambda_{iklm}$ should expand in the combination of products of components of n and isotropic tensors. A general form of such a tensor is

$$\lambda_{iklm}=\lambda_{1}n_{i}n_{k}n_{l}n_{l}+\lambda_{2}n_{i}n_{k}\delta_{lm}+\lambda_{3}\left(n_{i}n_{l}\delta_{km}+n_{i}n_{m}\delta_{kl}+n_{k}n_{l}\delta_{im}+n_{k}n_{m}\delta_{im}\right)$$

(A17) $$\;+\lambda_{4}n_{l}n_{m}\delta_{ik}+\lambda_{5}\delta_{ik}\delta_{lm}+\lambda_{6}\left(\delta_{il}\delta_{km}+\delta_{im}\delta_{kl}\right).$$

Substituting (A16) in (A17), one obtains for the magnetoelastic contribution

(A18) $$u_{\mathrm{em}}\;=\;-\;\sigma_{lm}\left[\lambda_{1}{\left(mn\right)}^{2}n_{l}n_{m}\;+\;2\lambda_{3}\left(n_{l}m_{m}\;+\;n_{m}m_{l}\right)\;+\;\lambda_{4}{\left(mn\right)}^{2}\delta_{lm}\;+\;2\lambda_{6}m_{l}m_{m}\right];$$

here the terms that do not comprise the components of m are omitted as they are not affected by the applied field.

Defining the strain tensor in the conventional way, one arrives at the expression

(A19) $$e_{ik}^{\left(\mathrm{strict}\right)}\;=\;-\;\frac{\partial u_{\mathrm{me}}}{\partial \sigma_{ik}}\;=\;\lambda_{1}{\left(mn\right)}^{2}n_{i}n_{k}\;+\;2\lambda_{3}\left(n_{i}m_{k}+n_{k}m_{i}\right)\left(mn\right)\;+\;\lambda_{4}{\left(mn\right)}^{2}\delta_{ik}\;+\;2\lambda_{6}m_{i}m_{k}.$$

In the initial state ($H_{0}=0$), where m‖n, i.e., $\left(mn\right)=1$, formula (A19) yields

(A20) $$e_{ik}^{\left(0\right)}\;=\;\left(\lambda_{1}\;+\;4\lambda_{3}\;+\;2\lambda_{6}\right)n_{i}n_{k}\;+\;\lambda_{4}\delta_{ik}.$$

This expression resembles the spontaneous striction that a ferri-/ferromagnet undergoes upon cooling below the Curie point. The applied field does not affect this contribution. As a result, the part of magnetostrictive strain, which is indeed orientationally dependent, is written as

$${\tilde{e}}_{ik}^{\left(\mathrm{strict}\right)}\;=\;e_{ik}^{\left(\mathrm{strict}\right)}\;-\;e_{ik}^{\left(0\right)}=\lambda_{1}\left[{\left(mn\right)}^{2}-1\right]n_{i}n_{k}\;+\;2\lambda_{3}n_{i}\left[\left(mn\right)m_{k}\;-\;n_{k}\right]+\;2\lambda_{3}n_{k}\left[\left(mn\right)m_{i}\;-\;n_{i}\right]\;$$

(A21) $$\;+\;\lambda_{4}\left[{\left(mn\right)}^{2}-1\right]\delta_{ik}+2\lambda_{6}\left(m_{i}m_{k}-n_{i}n_{k}\right).$$

In a single-domain particle in linear approximation with respect to parameter $H/H_{A}$, the relation between magnetization and applied field is established by Equation (2) of the main text; note that with the adopted accuracy one has (mn)=1. Substituting this in (A21) and omitting the tilde sign, one finds

(A22) $$e_{ik}^{\left(\mathrm{strict}\right)}\;=\;2\frac{H}{H_{A}}\left(\lambda_{3}+\lambda_{6}\right)\left[n_{i}h_{k}+n_{k}h_{i}-2n_{i}n_{k}\left(nh\right)\right],$$

so that magnetostriction is described by a single constant $\tilde{\lambda }=\lambda_{3}+\lambda_{6}$ which by its physical meaning should be identified with $\lambda_{s}$. As seen from (A22), in a field parallel to the anisotropy axis (h=n) the strain is zero, as intended.

## Appendix C. Piezoelectric Strain Response of Uniaxially Oriented Matrix

According to the basic theory [31], the strain that is due to the piezoeffect is

(A23) $$e_{ik}^{\left(\mathrm{piezo}\right)}=\gamma_{l;ik}E_{l}.$$

In the considered model it is assumed that as a result of poling the polymeric matrix is driven in a highly oriented—virtually single-domain—state characterized by unit vector ν that denotes the axis of piezoelectric anisotropy; to a certain extent ν is similar to the director of a nematic liquid crystal. We note that, although in reality, the degree of matrix orientation is never perfect, the degree of orientation of β phase in PVDF could be made rather high provided that electric poling is combined with mechanical processing [12,13,14].

Let vector ν be directed across the film. This enables one to define the piezoelectric tensor **γ** in the form

(A24) $$\gamma_{i;kl}=A\nu_{i}\delta_{kl}+B\nu_{i}\nu_{k}\nu_{l}+\frac{1}{2}C\left(\nu_{l}\delta_{ik}+\nu_{k}\delta_{il}\right),$$

where *A* to *C* are some material constants. In this representation and assuming that the Oz axis is aligned with ν, one has

(A25) $$e_{ik}^{\left(\mathrm{piezo}\right)}=\gamma_{l;ik}E_{l}=\left(\begin{array}{ccc} AE_{z} & 0 & \frac{1}{2}CE_{x}\\ 0 & AE_{z} & \frac{1}{2}CE_{y}\\ \frac{1}{2}CE_{x} & \frac{1}{2}CE_{y} & \left(A+B+C\right)E_{z} \end{array}\right).$$

Meanwhile, in the physics of piezoelectrics, a specific set of notations for piezocoefficients is in use. In particular, the stress-induced polarization is presented as

(A26) $$P_{i}=d_{ik}\;t_{k},$$

where the matrix of material parameters d has dimensions 3×6, and the 6D vector t is composed of the independent components of stress tensor with allowance for the symmetrical nature of the latter. On the other hand, the same relation has a general form [31]:

(A27) $$P_{i}=\gamma_{i;kl}\;\sigma_{kl}.$$

Comparison of formulas (A26) and (A27) enables one to find explicit relations between the components of tensor **γ** and the parameters $d_{ik}$. This transformation becomes yet simpler since in the coordinate frame with Oz directed along ν, matrix d in PVDF has just three non-zero components, viz., $d_{15}$, $d_{31}$ and $d_{33}$ [14,42]. With that, coefficients A÷C from (A24) expand as

(A28) $$A=d_{31},\;B=d_{33}-d_{15}-d_{31},\;C=d_{15}.$$

After substituting equalities (A28), expression (A25) assumes the form convenient for calculations:

(A29) $$e_{ik}^{\left(\mathrm{piezo}\right)}=\gamma_{l;ik}E_{l}=\left(\begin{array}{ccc} d_{31}E_{z} & 0 & \frac{1}{2}d_{15}E_{x}\\ 0 & d_{31}E_{z} & \frac{1}{2}d_{15}E_{y}\\ \frac{1}{2}d_{15}E_{x} & \frac{1}{2}d_{15}E_{y} & d_{33}E_{z} \end{array}\right).$$

It is worth noting that relations (A28) are of essential importance for any quantitative calculations since only the values for $d_{ik}$ are given in the papers and textbooks on the subject.
